# Development of an Aotearoa New Zealand adapted Mediterranean dietary pattern and Kai/food basket for the He Rourou Whai Painga randomised controlled trial

**DOI:** 10.3389/fnut.2024.1382078

**Published:** 2024-07-26

**Authors:** Anna Worthington, Eva Liu, Meika Foster, Summer Rangimaarie Wright, Fiona E. Lithander, Clare Wall, Rajshri Roy, Amber Parry-Strong, Jeremy Krebs, Andrea Braakhuis

**Affiliations:** ^1^Discipline of Nutrition, Faculty of Medical and Health Sciences, University of Auckland, Auckland, New Zealand; ^2^Edible Research Ltd., Ohoka, New Zealand; ^3^Liggins Institute, University of Auckland, Auckland, New Zealand; ^4^New Zealand National Science Challenge High Value Nutrition, Liggins Institute, University of Auckland, Auckland, New Zealand; ^5^Centre for Endocrine, Diabetes and Obesity Research (CEDOR), Wellington, New Zealand; ^6^Department of Medicine, University of Otago Wellington, Wellington, New Zealand

**Keywords:** Mediterranean diet, metabolic syndrome, cardiovascular diseases, Aotearoa, New Zealand, dietary pattern, dietary behaviour change

## Abstract

**Background:**

Following a Mediterranean diet (MedDiet) is associated with a lower risk of cardiovascular disease. He Rourou Whai Painga is a dietary intervention trial with behaviour change support that seeks to determine whether a MedDiet pattern can provide equivalent benefits in Aotearoa New Zealand (NZ), a country where cardiovascular disease is a leading cause of death. To do this, the MedDiet needs to be adapted in an acceptable way for NZ, with consideration of the Māori (indigenous) population.

**Methods:**

The MedDiet was defined using existing MedDiet scoring tools and adapted to the NZ context using local guidelines. The resulting NZ MedDiet pattern was used to develop a kai/food basket, including products from industry partners, for participants in He Rourou Whai Painga. Criteria set for the kai/food basket included providing up to 75% of energy requirements and falling within the Australia/NZ Acceptable Macronutrient Distribution Range to reduce risk of chronic disease. Māori researchers on the team provided support to ensure Mātauranga Māori (Māori knowledge and values) was upheld through this process.

**Results:**

The NZ MedDiet pattern criteria was similar to the identified MedDiet scoring tools, with differences in recommendations for dairy, red meat, alcohol and olive oil. The resulting kai/food baskets were estimated to provide on average 73.5% of energy requirements for households, with 36% from fat, 8.6% from saturated fat, 17% protein, and 42% carbohydrate. Forty-two industry partners, including 3 Māori businesses, agreed to provide 22 types of food products towards the total.

**Conclusion:**

Small, feasible changes to the MedDiet can be made to align with the NZ guidelines and food environment. However, this eating pattern still differs from what the population, particularly Māori, are currently consuming. Continued partnership with Māori and additional behavioural support is important to facilitate adherence to this dietary pattern within He Rourou Whai Painga.

**Trial registration**: https://www.anzctr.org.au/Default.aspx, identifier ACTRN12622000906752 and https://www.isrctn.com/, identifier ISRCTN89011056.

## 1 Introduction

Over the past two decades, the prevalence of many metabolic diseases has increased, presenting a significant global health burden (1, 2). In Aotearoa New Zealand (NZ), cardiovascular disease (CVD) is the leading cause of death, accounting for a third of all deaths annually (3), and approximately 5–6% of the population have type 2 diabetes mellitus (T2DM) (4). Over the past seven decades, mortality rates have been consistently higher for Māori (indigenous people of NZ), than non-Māori. Māori are 2.5 times more likely to have T2DM than non-Māori, and the prevalence of T2DM is the highest for Pacific people in NZ (5). Differences in prevalence and mortality signify the disproportionate and increased burden of cardiometabolic disease on Māori and Pacific peoples (4, 6). Metabolic syndrome (MetS) is a combination of multiple risk factors that increases the risk of CVD and T2DM (1). It is characterised by interconnected factors including hypertension, dyslipidaemia, disordered glucose metabolism, and obesity (2).

Unhealthy dietary habits and physical inactivity are important modifiable contributors to cardiometabolic disease risk worldwide (7, 8). The Mediterranean diet (MedDiet) is a well-studied dietary pattern characterised by an abundance of plant-based foods, olive oil, seafood, wholegrains and poultry, and low to moderate intake of red and processed meats, dairy products, and processed food (9, 10). Meta-analyses of the effects of the MedDiet on the MetS have demonstrated an overall positive impact of the MedDiet in decreasing MetS and its component factors in adults, including decreased blood glucose levels, blood pressure, and improved lipid profile (11), as well as decreasing the risk of overall mortality, CVD, and T2DM (12).

Considering the promise of the MedDiet in addressing MetS, it is of interest to investigate its potential in the New Zealand population. Indeed, the Eating and Activity Guidelines for NZ Adults (13) include the same food groups as the MedDiet, with only slight variations in the recommended proportions of different components (14). Additionally, aspects of the MedDiet align with traditional kai/food types and practices for Māori, such as a diet high in seafood (15), and valuing local, seasonal food, how it is prepared and the social context in which it is consumed (16). However, the 2008 NZ Adult Nutrition Survey reported that the average NZ diet differs significantly from the Eating and Activity Guidelines, and, in turn, a MedDiet. In particular, there was a higher intake of dairy and meat. This likely reflects food choices influenced by NZ’s history as an agricultural nation (17, 18). More recent cross-sectional studies of 908 (14) and 350 (19) NZ adults have demonstrated poor alignment with the MedDiet, indicating a significant shift in food choices will be required if the MedDiet is to be adopted in the NZ population (14, 19). Indeed, challenges exist with the transferability and adoption of the MedDiet to non-Mediterranean populations (20, 21); these include accessing and purchasing MedDiet foods such as olive oil (22), differences in habitual intake (14) and therefore adherence to a different dietary pattern (21), culture, religion, traditional cooking practices, and economic burden (23). Despite this, the MedDiet has been adapted and successfully implemented in different populations, such as in Northern Ireland and Australia, with concomitant improvements in MetS components and markers of cardiovascular risk (20, 24), demonstrating its potential for translation to other non-Mediterranean populations, such as New Zealand (25).

As there are substantial potential health benefits of the MedDiet, adaptation of the MedDiet to the NZ context should be explored, with consideration of the needs of Māori given the health disparities they experience. The He Rourou Whai Painga randomised controlled trial (RCT) aims to evaluate if a MedDiet pattern incorporating high quality NZ foods (“NZMedDiet”) improves a composite assessment of cardiometabolic health, the metabolic syndrome severity score (MetSSS) (26) for individuals identified as being at risk of cardiometabolic disease, in a study sample enriched for Māori. The planned intervention for this RCT is delivery in a household/whānau setting, underpinned by behavioural science. To support adherence to the NZMedDiet, participants will be provided kai/food baskets with high quality NZ foods that align with the NZMedDiet. Transparent documentation of the adaption or interpretation of a dietary pattern within nutrition trials is essential for providing context to observed results. However, the process of designing or adapting dietary patterns, such as the MedDiet, for free-living individuals who participate within trials is seldom reported (25). Consequently, the objective of the study reported here is to document the development of the NZMedDiet and composition of the kai/food baskets used in He Rourou Whai Painga; doing so provides a tangible process for adapting the MedDiet to other non-Mediterranean populations.

The present study aims to:

1. Adapt and define a MedDiet in the NZ context (“NZMedDiet”) for He Rourou Whai Painga.
2. Develop kai/food baskets that provide whānau/households with up to 75% of their estimated energy requirements with foods that align with the NZMedDiet in He Rourou Whai Painga.

## 2 Methods

He Rourou Whai Painga is a multi-centre trial consisting of two RCT phases and a longitudinal cohort study. The recruitment target is 200 participants with a MetSSS greater than 0.35 (26). Additionally, aligned both to the NZ Government’s Vision Mātauranga policy to support research of relevance to Māori and a collective worldview, a whānau-led approach will be taken where up to five members of a household in addition to the index participant will be invited to participate in the intervention. Full details on the trial have been published elsewhere (27). In brief, the first RCT compares the NZMedDiet to usual dietary intake with a primary outcome of the MetSSS 12 weeks after randomisation. For the 12-week intervention, participants will receive kai/food baskets containing ingredients and recipes for main meals and snacks, providing up to 75% of the energy requirements of the household/whānau, as per the protocol (27). Participants will also receive access to web-based nutrition education and opt-in online social support to help them adhere to the NZMedDiet. The second RCT compares the ongoing opt-in online social support, to no online social support with an outcome assessment 12-weeks after the second randomisation. From beginning the dietary intervention, the cohort will be followed for a total of 52 weeks.

**Figure 1:**
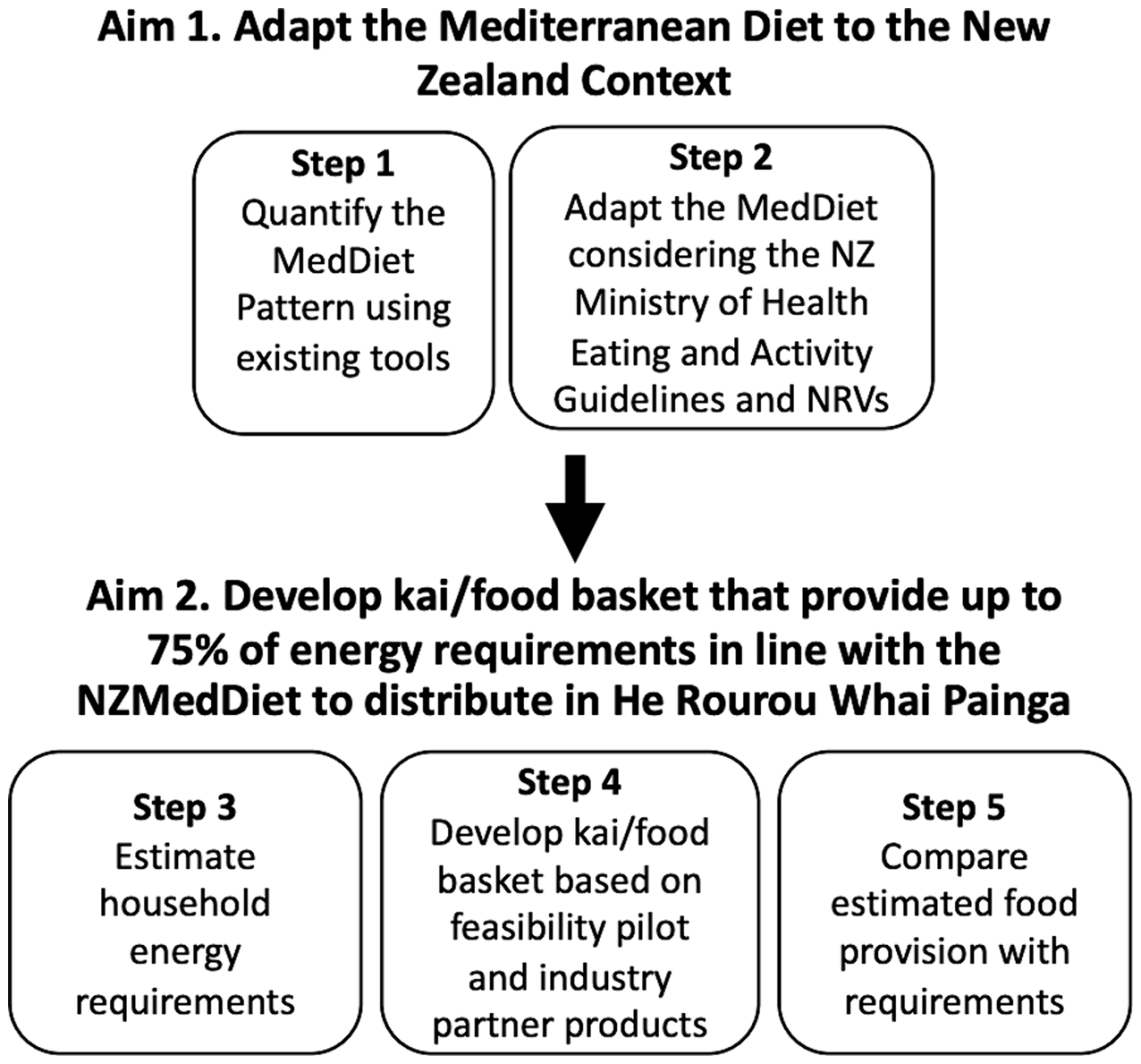
Process of developing kai/food baskets for He Rourou Whai Painga that align with a Mediterranean Diet (MedDiet) in the New Zealand (NZ) context. NRVs, Nutrient Reference Values for Australia and NZ (including the Acceptable Macronutrient Distribution Range to reduce risk of chronic disease); NZMedDiet, New Zealand Mediterranean Diet pattern.

A key foundation of this trial, therefore, was the development of the NZMedDiet and composition of the kai/food baskets to be received by all 200 participants and their participating whanau members. The process of developing this dietary pattern and kai/food baskets for He Rourou Whai Painga is outlined in Figure 1. This was overseen by a subgroup (the “NZMedDiet subgroup”) of study researchers consisting of eight NZ registered dietitians, two members of the consumer insights team, and another NZ registered dietitian from a commercial meal kit company. Importantly, Māori researchers were partnered with from the conception of this trial to support Pākehā/non-indigenous researchers with the design and implementation of this trial; this included advice while designing the dietary pattern to ensure its acceptability by Māori.

### 2.1 Adapting the MedDiet to the New Zealand context

#### 2.1.1 Step 1. Quantify the MedDiet pattern using existing tools

The recommended number and size of food group servings within a MedDiet varies throughout the literature (9, 28, 29). For the study reported here, the literature-based adherence score (LAS) to the Mediterranean diet (29) and the widely used Mediterranean Diet Adherence Screener (MEDAS) scoring tool (30) were used to determine the MedDiet composition, including the serve number and sizes for each food group. The LAS was developed by Sofi and colleagues using data from a meta-analysis of cohort studies investigating the association between adherence to the MedDiet and health outcomes (29). It comprises nine food categories, with a maximum score of 18 points indicating the highest adherence relative to the average of the general population (28). The MEDAS, developed by the Prevención con Dieta Mediterránea investigators (30), is a 14-item yes/no questionnaire and includes additional questions related to criteria characteristic of a MedDiet that the LAS does not contain. Adherence to the MedDiet measured using both tools has been associated with positive health outcomes (31, 32).

#### 2.1.2 Step 2. Adapt the MedDiet considering the NZ Ministry of Health eating and activity guidelines to produce a NZ MedDiet pattern

Two NZ registered dietitians from the NZMedDiet subgroup compared the recommended number of serves and portion sizes from the LAS and MEDAS tools to the NZ Ministry of Health Eating and Activity Guidelines (“the NZ guidelines”) (13), for the following foods and food groups: olive oil, vegetables, fruits, breads and cereals, legumes, nuts, fish/seafood, eggs, poultry, dairy foods, alcohol, and sweets. The NZ Heart Foundation guidelines were used to guide the recommendations for red meat (33). Portion sizes from the identified tools were adapted to match the NZ guidelines. Where a discrepancy existed between the number of serves recommended in the MedDiet versus the NZ guidelines, a consensus and justification were reached by the dietitians for adapting the NZMedDiet criteria to be feasible in the NZ setting, while still aligning with the principles underpinning the MedDiet; this was relevant for dairy foods, olive oil and alcohol. The output of this exercise was an objectively defined NZMedDiet. Additionally, for the purpose of He Rourou Whai Painga, it was determined that the NZMedDiet should closely align with the Acceptable Macronutrient Distribution Range (AMDR) for the reduction of chronic disease risk outlined in the Nutrient Reference Values for Australia and NZ (34).

### 2.2 Development of NZMedDiet Kai/food baskets for He Rourou Whai Painga

#### 2.2.1 Step 3. Estimating whānau/household energy requirements

In the RCT, food is provided to supply up to 75% of a whānau/household’s energy requirement through provision of kai/food baskets. Energy requirements for adults were based on the Australia NZ Food Standards Code 1.2.8, as derived from average intakes of adult males and females surveyed in Australia and NZ (35, 36), while the Nutrient Reference Values for Australia and NZ were used to estimate the energy needs of children aged 11–17 years and 5–10 years (34, 37). To ensure food provision to participating whānau/households of up to six people is consistent and workable, three versions of the kai/food baskets were designed for whānau/household sizes of two, four, and six members; energy estimates were calculated based on pragmatically assigned whānau/household makeups of two adults, three adults and one child (5-10 yr), and four adults and two children (5-10 yr), respectively. Of note, older children aged 11–17 years had the same energy requirements as adults, and consequently any ‘adult’ in the household makeup could also be a child aged 11–17 years.

#### 2.2.2 Step 4. Developing Kai/food baskets

The kai/food baskets for the trial are based on the following criteria: they must provide as close as possible to 75% of total energy; align with the NZMedDiet criteria (Table 1); be consistent with the AMDR for the reduction of chronic disease risk; include products from NZ food and beverage companies (industry partners), including Māori companies; and be feasible to assemble and deliver to whānau/households in three NZ regions both in the North and South Island of NZ. A feasibility trial of He Rourou Whai Painga was conducted at the Centre for Endocrine, Diabetes and Obesity Research (CEDOR) in Wellington and at Tū Kotahi Māori Asthma and Research Trust at Kōkiri Marae (a community-based traditional Māori meeting place) in Lower Hutt, Wellington. This involved providing participants with up to 75% of their energy needs from foods aligning with a MedDiet. The feasibility trial confirmed that the use of commercial companies for the distribution of kai/food baskets was feasible and acceptable to participants (38). Consequently, two commercial companies were selected for the provision of kai/food baskets in the main trial, one of which is a meal kit home delivery service, and the other is an online grocery provider.

**Table 1:** Foods and their recommended serves per participant for the New Zealand Mediterranean Diet Pattern (NZMedDiet) compared to the literature-based adherence score, and Mediterranean Diet Adherence Screener (MEDAS) scoring tool.

	NZMedDiet		Literature-based adherence score		MEDAS tool	
Foods	Recommended serves per day/week/meal	Serve size	Recommended serves per day/week/meal	Serve size	Recommended serves per day/week/meal	Serve size
Olive oil	≥ 2 serves / day	15 mL	*Regular use*	*Not defined*	≥ 4 serves / day	15 mL
Vegetables	≥ 400 g / day		>2.5 serves / day	100 g	≥ 400 g / day	
Fruits	≥ 3 serves / day	150 g	>2 serves / day	150 g	≥ 3 serves / day	*Not defined*
Breads and cereals	1–2 serves / meal	*	>1.5 serves / day	130 g	*Not included*	
Legumes	≥ 3 serves / week	70 g^$^	>2 serves / day		≥ 3 serves / week	50 g
Nuts	≥ 3 serves / week	30 g	*Not included*		≥ 3 serves / week	30 g
Fish/seafood	≥ 3 serves / week	115 g raw	>2.5 serves / week	100 g	≥ 3 serves / week	100-150 g
Eggs	2–4 serves / week	1 egg	*Not included*		*Not included*	
Poultry	≥ 2 serves / week	100 g raw	*Comes under meat*		*Preferential consumption of white meat*	
Dairy foods	2 serves / day	1 cup^%^	<1 serve / day	180 g	*<1 serve/day (of butter, margarine, or cream)*	12 g
Red meat	≤1 serve / day	62.5 g raw	<1 serve / day	80 g	<1 serves / day	150 g
Sweets	≤ 1 serve / week		*Not included*		< 3 serves / day	1 pastry
Alcohol	*Not included*		1–2 Alcohol unit (AU) / day	1 AU = 12 g	≥ 7 glasses / week	

*1 slice (40 g) wholegrain bread; ½ medium (40 g) wholegrain roll or flat bread; ½ cup (75–120 g) cooked rice, pasta, noodles, barley, buckwheat, semolina, polenta, bulgur or quinoa; ½ cup (120 g) cooked porridge; ¼ cup (30 g) muesli; 2 breakfast wheat biscuits; 2/3 cup cereal flakes (wholegrain where possible); 3 (35 g) crispbreads or crackers (wholegrain where possible). ^$^cooked or canned. ^%^NZ MoH define as: standard serve (500–600 kJ) is about the same as: 1 cup (250 mL) low or reduced fat fresh, UHT long life, reconstituted powdered milk or buttermilk; 2 slices (40 g) or a 4 × 3 × 2 cm piece of cheese such as Edam ¾ cup (200 g) low- or reduced-fat yoghurt; 1 cup (250 mL) calcium-fortified plant based milk alternatives (e.g., soy, rice, almond, oat milk; with at least 100 mg of added calcium per 100 mL).

The meal kit home delivery service is responsible for providing a weekly dinner box and fruit box. Dinner boxes are delivered to participants’ homes with recipe cards and all components of those meals appropriate to the household size; to serve either two, four, or six people. Researchers are responsible for selecting dinner meals in advance for participants from those offered each week by the meal kit home delivery service; the NZMedDiet subgroup established selection criteria to ensure meals chosen aligned with the NZMedDiet. The online grocery provider is responsible for the distribution of a weekly grocery box, which contains foods for breakfasts, lunches, and snacks. Additionally, a one-off starter box of food products provided by industry partners is distributed by the grocery provider in the first week of participants beginning in the intervention.

Participant feedback from the feasibility trial was used to make decisions regarding the kai/food baskets for the main trial; of note, the feasibility trial provided a strong voice for populations facing higher disparities of CVD, with 55 and 13.8% of participants identifying as Māori and Pacific, respectively (38). Feedback highlighted a need for variety in breakfasts and lunch options, hence three variations of the grocery box were developed for the whānau/household sizes of two, four and six members, resulting in nine versions of the grocery box. Additionally, food items that were not liked were swapped for items favoured by the feasibility trial participants, or the quantity provided was reduced. Feasibility trial participants identified food items that differed from what they traditionally use, and the need for instruction on how to prepare them; hence ingredient lists, recipes and meal plans for the grocery boxes were developed by the NZMedDiet subgroup and made available on a participant-facing website.

Food provided in the kai/food baskets comes from two sources; food provided in-kind to the study by industry partners and food purchased by the research team. A list of potential industry partners, including Māori food businesses, was generated using information provided by the Directorate of the High Value Nutrition National Science Challenge and commercial knowledge of the research team. Each potential partner was contacted with details of the study and invited to attend online and in-person information sessions, before being invited to complete a business case template for in-kind provision of a product to the study. Offered products were then screened by the NZMedDiet subgroup and wider research team, and those that aligned with the study values and prespecified criteria were accepted and incorporated into the kai/food baskets. Diet-specific criteria that products were required to meet before being accepted into the trial included alignment with a MedDiet pattern, alignment with healthy food guidelines when consumed in the prescribed quantities (for example carbonated beverages were generally excluded even when low in sugar content) and were not dietary supplements.

A nutrition database containing foods available in NZ, FoodWorks (version 10, Xyris Pty Ltd), was used to analyse the nutrition content of the grocery, fruit, and starter boxes. An average estimate of the energy content of dinner meals was provided by the meal kit home delivery service dietitian. The total amount of energy predicted to be provided over the 12 weeks was compared by two NZMedDiet subgroup dietitians (independently, with any differences resolved by consensus) to the estimated energy requirements per whānau/household to ensure it was close to but did not exceed 75%.

#### 2.2.3 Step 5. Comparing estimated food provision with energy requirements

An estimate of energy intended to be provided by the kai/food baskets was compared to the amount of food required to meet the NZMedDiet criteria and total energy over 12 weeks. The number of serves of each food group per person provided over the 12 weeks was estimated based on the expected ingredients in the grocery, fruit and starter boxes, and as well as an average of 3 weeks of dinner recipes from the meal kit home delivery service. Of note, the dinner meals will differ every night across the 12 weeks in the actual study. The predicted nutrition content of the food provided was also compared to the AMDR for the reduction of chronic disease risk. Where possible, quantities of food intended to be provided were adjusted to more closely align with the AMDRs while still aligning with the NZMedDiet.

## 3 Results

### 3.1 Adapting the Mediterranean dietary pattern to the New Zealand context

#### 3.1.1 Step 1 and 2. Quantify the components of the NZ MedDiet pattern using existing tools

Table 1 depicts the recommended number and size of serves for the NZMedDiet compared to the ideal serve number and size in the LAS and MEDAS tool. Differences are apparent between the recommended number and size of food group serves in the LAS, MEDAS and NZ Guidelines for alcohol, red meat, dairy, and olive oil and were reconciled, as described below (13, 29, 30).

Portion sizes on the original LAS are derived from the calculation of mean value of weighted medians (or means) from the included cohort studies (29). Most of these aligned with the recommended serve sizes and numbers from the NZ guidelines, except for dairy and red meat. The NZ guidelines recommend 2.5–4 serves of dairy per day, depending on age and gender, where a serve is 250 mL of milk, 40 g cheese, or 200 g yoghurt (13). The LAS recommends less than one serve of dairy per day, where a serve is 180 g regardless of the source of dairy. Consensus was reached by the dietitians within the NZMedDiet subgroup to recommend two serves of dairy per day, which is at the conservative end of the NZ guidelines, to acknowledge the low to moderate consumption of dairy encouraged by the MedDiet. Additionally, the daily serve size of red meat was lowered from 80 g (LAS tool) and 150 g (MEDAS tool) to align with the NZ Heart Foundation guidelines of 62.5 g (Table 1), as per the evidence for reducing the occurrence of heart disease (33).

The NZ Guidelines do not provide specific recommendations on olive oil consumption (13). Additionally, quantifiable definitions were not provided for the use of olive oil in the LAS. Only one reference within the LAS included high olive oil intake as a component of the adherence score (39), and consequently these tertiles were used to define regular use (≥2 serves/day), frequent use (1–1.9 serves/day), and occasional use (<1 serve/day), where a serve is 15 mL (1 Tbsp). Although 1 point is awarded in the MEDAS tool for consuming ≥4 tbsp of olive oil per day, the LAS aligns more closely with the NZ context and was thought to be more acceptable and achievable given the primary sources of total and saturated fat, poly- and mono-unsaturated fatty acids in the NZ diet are butter and margarine (17), as well as considering the AMDRs where no more than 35 and 10% of energy from fat and saturated fat, respectively, is recommended.

Contrary to the MedDiet, the NZ Ministry of Health recommends limiting alcohol consumption to no more than two and three standard drinks per day for women and men, respectively, and including two alcohol-free days a week (40). Given the context of the study was to reduce risk of cardiovascular disease, the subgroup decided that explicit guidance about alcohol intake would not be given in He Rourou Whai Painga unless requested. If asked, participants would be referred to the NZ guidelines surrounding alcohol consumption.

### 3.2 Development of Kai/food baskets

#### 3.2.1 Step 3. Estimating whānau/household energy requirements

Energy requirements of the average adult and children aged 11–17 years were based on the recommended total energy intake of 8,700 kJ per day (34, 37). The mean energy requirement of boys and girls aged 5–10 years was estimated to be 6,900 kJ per day (34). Therefore, over a 12-week period it was estimated that a whānau/household of two, four and six members would be provided 1,096 MJ, 2097 MJ and 3,062 MJ to reach 75% of energy requirements, respectively (Table 2).

**Table 2:** Energy provided by kai/food baskets over 12 weeks compared to estimated whānau/household energy needs.

	2 Person Household (2 adults)	4 Person Household (3 adults +1 child; 5-10 yr)	6 Person Household (4 adults +2 children; 5-10 yr)
Grocery box 1 (kJ)	296,068	455,656	733,320
Grocery box 2 (kJ)	199,660	329,080	529,504
Grocery box 3 (kJ)	127,016	276,328	364,688
Fruit Box (kJ)	93,889	281,667	375,556
Dinner Box (kJ)	307,087	614,174	921,262
Starter Box (kJ)	63,581	63,581	63,581
Total energy provided (kJ)	1,087,238	2,020,423	2,987,847
100% Estimated energy requirements (kJ)	1,461,600	2,772,000	4,082,400
75% Estimated energy requirements (kJ)	1,096,200	2,079,000	3,061,800
Total household requirements provided (%)	74.4	72.9	73.2

#### 3.2.2 Step 4. Developing Kai/food baskets

**Figure 2:**
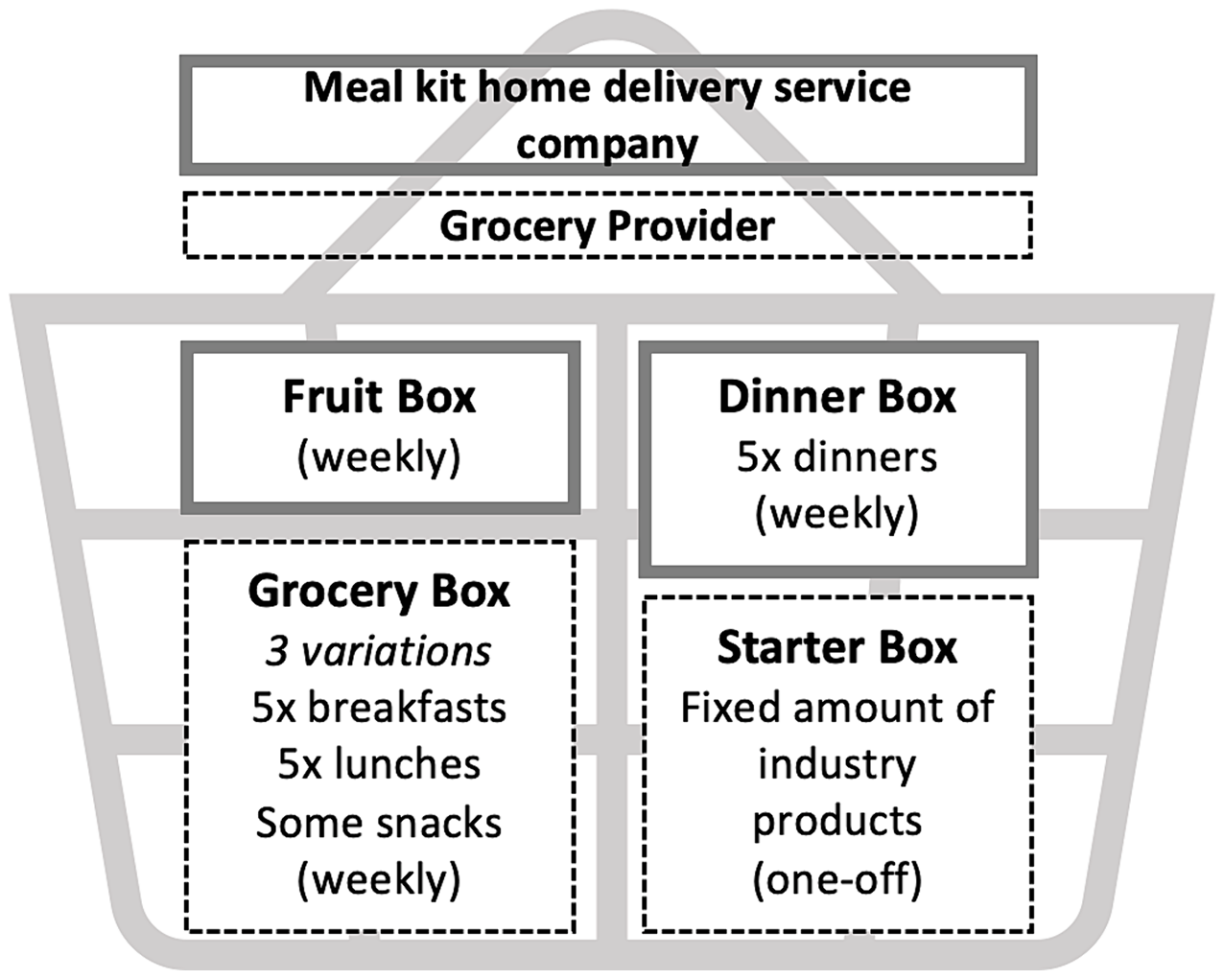
Kai/food baskets that whānau/households will be delivered by two providers in the phase one RCT intervention arm of He Rourou Whai Painga.

Figure 2 gives an overview of the kai/food basket that was developed. Identical to the feasibility trial, the meal kit home delivery service provides five dinner meals each week per participant in the main study, with each meal on average providing 2,559 kJ (example meals provided in Supplementary file 1). To ensure these dinner meals align with the NZMedDiet, the research team will use the following selection criteria each week: select as many fish-based meals as are available; as many vegetarian or chicken meals as are available; after that, choose a red meat-based meal that contains at least two serves of vegetables and uses wholegrains/legumes. Approximately 22 pieces of fruit are supplied per fruit box, with one, three and four boxes provided for whānau/households of two, four and six people, respectively. The kai/food baskets can be tailored to dietary preferences, such as vegetarian. The grocery box provides ingredients for approximately five breakfasts and five lunches per week; ingredient quantities and sample menus for each box can be found in Supplementary file 1. The quantity of additional snacks is based on providing up to 75% of total energy per whānau/household. The dinner, grocery, fruit and starter boxes are estimated to provide an average of 73.5% of a whānau/household’s total energy requirements for the specific whānau/household (Table 2).

In total, 42 industry partners, three of which are Māori food businesses, will provide 22 different products, such as macadamias, kiwifruit, and seafood (Supplementary file 2). Of these partners, 18 will provide olive oil, and 9 will provide lean red meat products.

#### 3.2.3 Step 5. Comparing estimated food provision with energy requirements

Compared to the NZMedDiet, the kai/food baskets are estimated to provide 100% or more of requirements for fruit, legumes, nuts, poultry and eggs, between 50 and 75% of vegetables, red meat, and breads and cereals, while less than 50% of olive oil, fish and dairy foods would be provided (Table 3).

**Table 3:** Amount of food required to meet the NZMedDiet criteria over 12 weeks compared to an estimate of what will be provided by the He Rourou Whai Painga kai/food baskets.

Foods	Units	100% amount required pp. for 12 weeks	Estimated total amount provided by boxes per person over 12 weeks^*^	Percentage of NZMedDiet recommendations provided
Olive oil	L	2.52	1.0	39.7
Vegetables	Kg	33.6	24.6	73.3
Fruits^%^	Kg	37.8	38.7	102.4
	pieces	252	258.0	102.4
Breads and cereals	Serves^$^	504	289.0	57.3
Legumes	Kg	2.52	4.1	161.6
Nuts	Kg	1.08	1.4	132.8
Fish/seafood	Kg	4.14	1.7	40.6
Eggs	eggs	24–48	36.0	100
Poultry	Kg	2.4	3.6	150
Dairy foods	Serves^&^	168	58.1	34.6
Red meat	Kg	5.25	2.9	55.2
Sweets	NA	0	*Not provided*	0.0
Alcohol	NA	0	*Not provided*	0.0

$ 1 slice (40 g) wholegrain bread; ½ medium (40 g) wholegrain roll or flat bread; ½ cup (75–120 g) cooked rice, pasta, noodles, barley, buckwheat, semolina, polenta, bulgur or quinoa; ½ cup (120 g) cooked porridge; ¼ cup (30 g) muesli; 2 breakfast wheat biscuits; 2/3 cup cereal flakes (wholegrain where possible); 3 (35 g) crispbreads or crackers (wholegrain where possible).

& 1 cup (250 mL) low or reduced fat fresh, UHT long life, reconstituted powdered milk or buttermilk; 2 slices (40 g) or a 4 × 3 × 2 cm piece of cheese such as Edam ¾ cup (200 g) low- or reduced-fat yoghurt; 1 cup (250 mL) calcium-fortified plant based milk alternatives (e.g., soy, rice, almond, oat milk; with at least 100 mg of added calcium per 100 mL).

* Calculations based on the amounts provided in the grocery boxes for a 4 person household.

% Dried fruits were not counted towards serves of fruit in this analysis.

The kai/food baskets that provide 75% of total energy are predicted to be slightly lower for carbohydrate and higher for fat than the macronutrient distribution to reduce chronic disease risk, while meeting the recommendations for protein and saturated fat (Table 4).

**Table 4:** Acceptable macronutrient distribution ranges to reduce chronic disease risk and predicted provision by He Rourou Whai Painga kai/food baskets.

Nutrient	Lower end of recommended intake range	Upper end of recommended intake range	Average predicted % provided by kai/food baskets*
Protein	15% of energy	25% of energy	17%
Total Fat	20% of energy	35% of energy	39%
Saturated fat	NA	10% of energy	8.6%
Carbohydrate	45% of energy	65% of energy	44%

*Excluding starter box as it provides negligible energy over the 12 weeks.

## 4 Discussion

This study reports the process by which the investigators of He Rourou Whai Painga created the NZ version of the MedDiet, and produced a method to deliver foods to participants and their whānau/households aligning with this dietary pattern to meet 75% of their energy needs. The NZMedDiet was developed by adapting the MedDiet, as defined by the LAS and MEDAS tools, to the NZ context and guidelines. The NZMedDiet criteria were used to guide the development of kai/food baskets, providing participants and household members in He Rourou Whai Painga with an estimated average of 73.5% of energy for 12 weeks. Kai/food baskets include NZMedDiet ingredients for breakfasts, lunches, dinners, and snacks, including 22 products from 42 industry partners.

The premise of promoting consumption of the MedDiet is that it will confer health benefits, such as reduction in CVD risk factors (11, 12). Consequently, the degree of difference between the NZMedDiet and the traditional MedDiet needs to be considered. Although the NZMedDiet does not exactly match any previously defined MedDiet scoring tool, when compared to the eight MedDiet questionnaires reviewed by Chiriacò et al. (28), all food items in the NZMedDiet fall within the identified ranges of existing recommended intakes. This indicates that the NZMedDiet is within the scope of how the MedDiet can plausibly be defined. However, when compared to the PyrMDS, considered the most accurate and reliable tool to estimate MedDiet adherence (28), the NZMedDiet falls just under the recommendations for vegetables (≥400 g/day NZMedDiet versus ≥480 g/day PyrMDS), legumes (210 g/week versus 300 g/week), and nuts (90 g/week versus 30-60 g/day), while recommending more red meat (≤437.5 g/week versus ≤200 g/week), and fewer sweets (≤1 serve/week versus ≤2/week) (28, 41). Additionally, the lifestyle habits of Mediterranean populations, such as physical activity, pattern of meals, and context in which meals are consumed, may act as confounding factors to the observed health benefits (22). However, a particular strength of He Rourou Whai Painga is its whānau/household collective wellbeing approach in recognition that food is largely consumed in a shared environment and it is common for cardiometabolic risk to cluster within a family (42); therefore, dietary interventions that target the wider family may be more likely to be successful compared to those which focus solely on improving the health of an individual. These differences in the NZMedDiet criteria compared to the traditional MedDiet and additional lifestyle considerations will be important to consider when analysing the effect of the NZMedDiet in He Rourou Whai Painga.

Trying to fit a MedDiet-style pattern to the AMDRs proved challenging with the provided kai/food baskets predicted to exceed the desired AMDR for fat (39%) and fall short of that for carbohydrates (44%). This is not surprising given the usual amount of fat in the MedDiet is 30–45% (9, 43). However, the type of fat may be of more importance in this case; the MedDiet typically has a higher ratio of mono- and poly-unsaturated fatty acids to saturated fatty acids which can improve LDL cholesterol levels, contributing to the reduction in CVD risk (44). The NZMedDiet is likely to have a similar ratio, as demonstrated by saturated fat predicted to provide less than 10% of total energy. The AMDR estimates do not account for the extra 25% of energy that participants are responsible for purchasing. Consequently, dietary assessment during the trial will be important to establish the actual macronutrient intake compared to the AMDR. Methods of measuring dietary intake will include the Otago Short Form Food Frequency Questionnaire and 24-h recalls (27).

To establish whether the NZMedDiet confers health benefits similar to the MedDiet it is key that participants are supported to adhere to it and that adherence is measured, as the level of adherence to a dietary pattern impacts the effect size on health outcomes (45, 46). For example, to achieve the desired NZMedDiet participants would ideally supplement the kai/food baskets with more olive oil, fish, vegetables and some bread and cereals, red meat, and dairy products. Given the current dietary pattern of NZ adults, particularly those of Māori and Pacific ethnicity, differs greatly from the MedDiet (14, 47) behavioural support to encourage purchase and consumption of these foods is important. Implementing a Mediterranean diet pattern in a non-Mediterranean country has its challenges, with previous research stating the following barriers to adopting such a diet; stress and work pressure; difficulties purchasing food items; increase in food costs and country-specific culture and climate being non-conducive (48). It will be important to identify specific barriers and effective support strategies relevant to the NZ context, including accounting for food practices (21, 49). To do this, the Behaviour Change Wheel (50) will be used in He Rourou Whai Painga, along with continued partnership with Māori as implemented in the feasibility trial (38). This behaviour change framework has been previously used to successfully develop a peer-support intervention to encourage dietary behaviour change towards a MedDiet in non-Mediterranean adults at high CVD risk (20, 51).

Strengths of this study include that we have clearly defined the recommended number of serves and portion sizes for each food group, enabling adherence to the dietary behaviours required within the NZMedDiet to be assessed, a factor frequently overlooked in dietary intervention trials (52, 53). Additionally, the involvement of Tū Kotahi Māori Asthma and Research Trust enabled the target audience to have a voice in what the kai/food baskets contained, demonstrating how these types of projects can be run in partnership with Māori in the community. Limitations of the design of the kai/food baskets include that they are designed based on fixed whānau/household makeups; three-person and five-person households will receive two-person and four-person kai/food baskets, meaning they are supplied less energy relative to other households of two-, four- and six-persons, to not exceed 75% of energy requirements. Additionally, restricting to six whānau/household members may exclude larger whānau/households from participating. The estimations of energy intake in this article are also limited by using 15 meals from the meal kit home delivery service over the 12 weeks; a greater variety of meals will be provided in the actual study. This estimation cannot be directly extrapolated to a two- or six-person whānau/household as, given the different energy requirements within the described whānau/households, the quantity of products received are not always factors of each other. For instance, a four-person whānau/household does not receive exactly double that of the two-person whānau/household, as its energy requirements are not double considering it factors in a child with lower energy requirements as a member. Again, this reinforces the importance of capturing dietary intake during the trial.

In summary, the MedDiet was able to be adapted to the NZ context for the purpose of He Rourou Whai Painga, along with the development of kai/food baskets containing locally sourced produce. He Rourou Whai Painga will provide valuable insight into the acceptability and effectiveness of this dietary pattern in the NZ population, particularly for Māori who experience a disproportionate and increased burden of CVD. Given the current NZ diet differs largely from the MedDiet, behavioural support and continued partnership with Māori will be important in facilitating adherence to the NZMedDiet.

## Data availability statement

The raw data supporting the conclusions of this article will be made available by the authors, without undue reservation.

## Ethics statement

Ethical approval was granted by the New Zealand Health and Disability Ethics Committee—Northern B branch—reference 2022 FULL 12045 for He Rourou Whai Painga. Consent is not applicable for this aspect of the trial.

## Members of the He Rourou Whai Painga Consortium

Jeremy D. Krebs, Department of Medicine, University of Otago Wellington, Wellington, New Zealand, Centre for Endocrine, Diabetes and Obesity Research (CEDOR), Wellington Regional Hospital –Te Whatu Ora, Wellington New Zealand, Principal investigator; Richard Gearry, Department of Medicine, University of Otago Christchurch, Christchurch, New Zealand; Troy L. Merry, Discipline of Nutrition, School of Medical Sciences, The University of Auckland, Auckland, New Zealand, Maurice Wilkins Centre for Molecular Biodiscovery, The University of Auckland, Auckland, New Zealand; Andrea Braakhuis, Discipline of Nutrition, School of Medical Sciences, The University of Auckland, Auckland, New Zealand; Fiona Lithander, Discipline of Nutrition, School of Medical Sciences, The University of Auckland, Auckland, New Zealand, Liggins Institute, The University of Auckland, Auckland, New Zealand; Meika Foster, Liggins Institute, The University of Auckland, Auckland, New Zealand, Edible Research Ltd, Ohoka, New Zealand; Anna Rolleston, Manawaora Integrated Health and Research Ltd, Tauranga, New Zealand; Amber Parry-Strong, Centre for Endocrine, Diabetes and Obesity Research (CEDOR), Wellington Regional Hospital–Te Whatu Ora, Wellington New Zealand; Cecilia Ross, Centre for Endocrine, Diabetes and Obesity Research (CEDOR), Wellington Regional Hospital –Te Whatu Ora, Wellington New Zealand; Mark Weatherall, Department of Medicine, University of Otago Wellington, Wellington, New Zealand; Denise Conroy, Plant and Food Research, Auckland, New Zealand; Cheryl Davies, Tū Kotahi Māori Asthma and Research Trust, Kōkiri Marae, Lower Hutt, New Zealand; Anna Worthington, 4 Discipline of Nutrition, School of Medical Sciences, The University of Auckland, Auckland, New Zealand.

## Author contributions

AW: Conceptualization, Data curation, Formal analysis, Methodology, Writing – original draft, Writing – review & editing. EL: Writing – original draft, Writing – review & editing. MF: Conceptualization, Data curation, Funding acquisition, Investigation, Methodology, Supervision, Writing – review & editing. SW: Data curation, Formal analysis, Methodology, Validation, Writing – review & editing. FL: Conceptualization, Formal analysis, Funding acquisition, Investigation, Methodology, Project administration, Resources, Writing – review & editing. CW: Formal analysis, Supervision, Writing – review & editing. RR: Data curation, Formal analysis, Writing – review & editing. AP-S: Data curation, Formal analysis, Funding acquisition, Investigation, Methodology, Writing – review & editing. JK: Conceptualization, Funding acquisition, Supervision, Writing – original draft, Writing – review & editing. AB: Conceptualization, Data curation, Formal analysis, Funding acquisition, Investigation, Methodology, Supervision, Validation, Writing – original draft, Writing – review & editing.

## Glossary

AMDR: Acceptable Macronutrient Distribution Range
AUSDRISK: Australian Type 2 Diabetes Risk Assessment Tool
COM-B: Capability Opportunity Motivation Behaviour model
CVD: Cardiovascular Disease
CEDOR: Centre for Endocrine, Diabetes and Obesity Research
LAS: Literature-Based Adherence Score
MEDAS: Mediterranean Diet Adherence Screener
MedDiet: Mediterranean Diet
MetS: Metabolic Syndrome
MetSSS: Metabolic Syndrome Severity Score
NZ: New Zealand
NZ Guidelines: New Zealand Ministry of Health Eating and Activity Guidelines
NZMedDiet: New Zealand Mediterranean Diet
PyrMDS: Pyramid based Mediterranean Diet Score
T2DM: Type 2 Diabetes Mellitus
Aotearoa: New Zealand
Kai: food
Māori: indigenous people of New Zealand
Mātauranga: traditions, values, and knowledge from a Māori world view
Whānau: family
