# Supplementary material for: Development of an Aotearoa New Zealand adapted Mediterranean dietary pattern and Kai/food basket for the He Rourou Whai Painga randomised controlled trial
Source: Front Nutr. 2024 Jul 26;11:1382078. doi: 10.3389/fnut.2024.1382078 (PMC11311200; doi:10.3389/fnut.2024.1382078)

**Supplemetary file 1.** Grocery box ingredients received by participants during the He Rourou Whai Painga trial are listed below. Participants received a total of 4 of each box across the 12 weeks (1 per week, on a 3 week cycle). Below ingredient lists are the ‘sample menus’ that participants were provided. These detail meal ideas they could make from the provided ingredients each week; recipes for the suggested meal ideas were also provided. An example of what a weeknight of meals could look like from the provider is also given.

**Box 1**

| Name | Description | Quantity |  |  |  |
| --- | --- | --- | --- | --- | --- |
|  |  | **2 person** | **4 person** | **6 person** |  |
| Weet-Bix | 375g box | 1 | 1 | 1 |  |
| Reduced fat milk | 2L | 1 | 2 | 3 |  |
| Sunflower seeds | 300g | 0 | 0 | 2 |  |
| Sesame seed rice cakes | 130g | 1 | 2 | 3 |  |
| Olive oil | 500mL | 2 | 2 | 3 |  |
| Multigrain wraps | 6 pack | 1 | 2 | 3 |  |
| Tuna in spring water | 185g tin | 1 | 2 | 3 |  |
| Mediterranean mix | 215g tub | 1 | 2 | 3 |  |
| Walnut pieces | 150g packet | 1 | 2 | 3 |  |
| Sultanas | 150g packet | 1 | 2 | 3 |  |
| Spinach | 300g packet | 1 | 2 | 3 |  |
| Wholegrain quinoa | 400g packet | 1 | 2 | 3 |  |
| Beans and lentils with wholegrains | cans | 2 | 4 | 6 |  |
| Red onion 3pc | 3pc | 1 | 2 | 3 |  |
| Cherry tomatoes | 250g punnet | 2 | 4 | 6 |  |
| NZ basil pesto | 150g packet | 1 | 2 | 2 |  |
| Kiwi fruits | Individual fruit | 10 | 20 | 30 |  |

**Box 2**

| Name | Description | Quantity |  |  |  |
| --- | --- | --- | --- | --- | --- |
|  |  | **2 person** | **4 person** | **6 person** |  |
| Breakfast cereal | 460g | 1 | 2 | 3 |  |
| Reduced fat milk | 2L | 1 | 2 | 3 |  |
| Multigrain bread | 700g loaf | 1 | 2 | 3 |  |
| Peanut butter | 500g | 1 | 1 | 2 |  |
| Green beans | 500g | 1 | 2 | 3 |  |
| Four bean mix | can | 2 | 4 | 6 |  |
| Beans, chickpeas and oats | can | 1 | 2 | 3 |  |
| Capsicum (any colour) | 1 | 1 | 2 | 3 |  |
| Red onion 3pc | 3pc | 1 | 2 | 3 |  |
| Tuna pocket (any flavour) | 110g | 1 | 2 | 3 |  |
| Organic parsley | 8g | 1 | 2 | 3 |  |
| Iceberg lettuce | 1 | 1 | 1 | 1 |  |
| Avocado | 1 | 1 | 2 | 3 |  |
| Hummus (pumpkin and kumara) | 380g | 1 | 1 | 2 |  |
| Vitaweat supersedes crackers | 250g | 1 | 2 | 3 |  |
| Kiwifruits | individual kiwi fruits | 10 | 20 | 30 |  |

**Box 3**

| Name | Description | Quantity |  |  |  |
| --- | --- | --- | --- | --- | --- |
|  |  | **2 person** | **4 person** | **6 person** |  |
| Eggs | 12 pack | 1 | 2 | 3 |  |
| Spinach 120g | 120g bag | 1 | 2 | 3 |  |
| Brown onion 3 pc | 3 pc | 2 | 2 | 6 |  |
| White button mushrooms | 200g punnet | 1 | 2 | 3 |  |
| Lite soy milk | 1L | 1 | 2 | 3 |  |
| Multigrain bread | 700g loaf | 2 | 3 | 3 |  |
| Iceberg lettuce | 1 | 1 | 2 | 2 |  |
| supie grocer avocado | 1 | 2 | 4 | 4 |  |
| Hummus (pumpkin and kumara) | 380g | 1 | 2 | 3 |  |
| Tuna pocket (any flavour) | 110g | 1 | 2 | 3 |  |
| Taco Tortillas 12 small | packet | 1 | 2 | 3 |  |
| Nacho spice mix | packet | 1 | 2 | 3 |  |
| Beef or lamb meat | 500g | 1 | 2 | 3 |  |
| Capsicum (any colour) | 1 | 1 | 2 | 3 |  |
| Tomatoes chopped in juice | 400g can | 1 | 2 | 3 |  |
| Red kidney beans | 400g can | 1 | 2 | 3 |  |
| Kiwifruit | individual kiwi fruits | 10 | 20 | 30 |  |


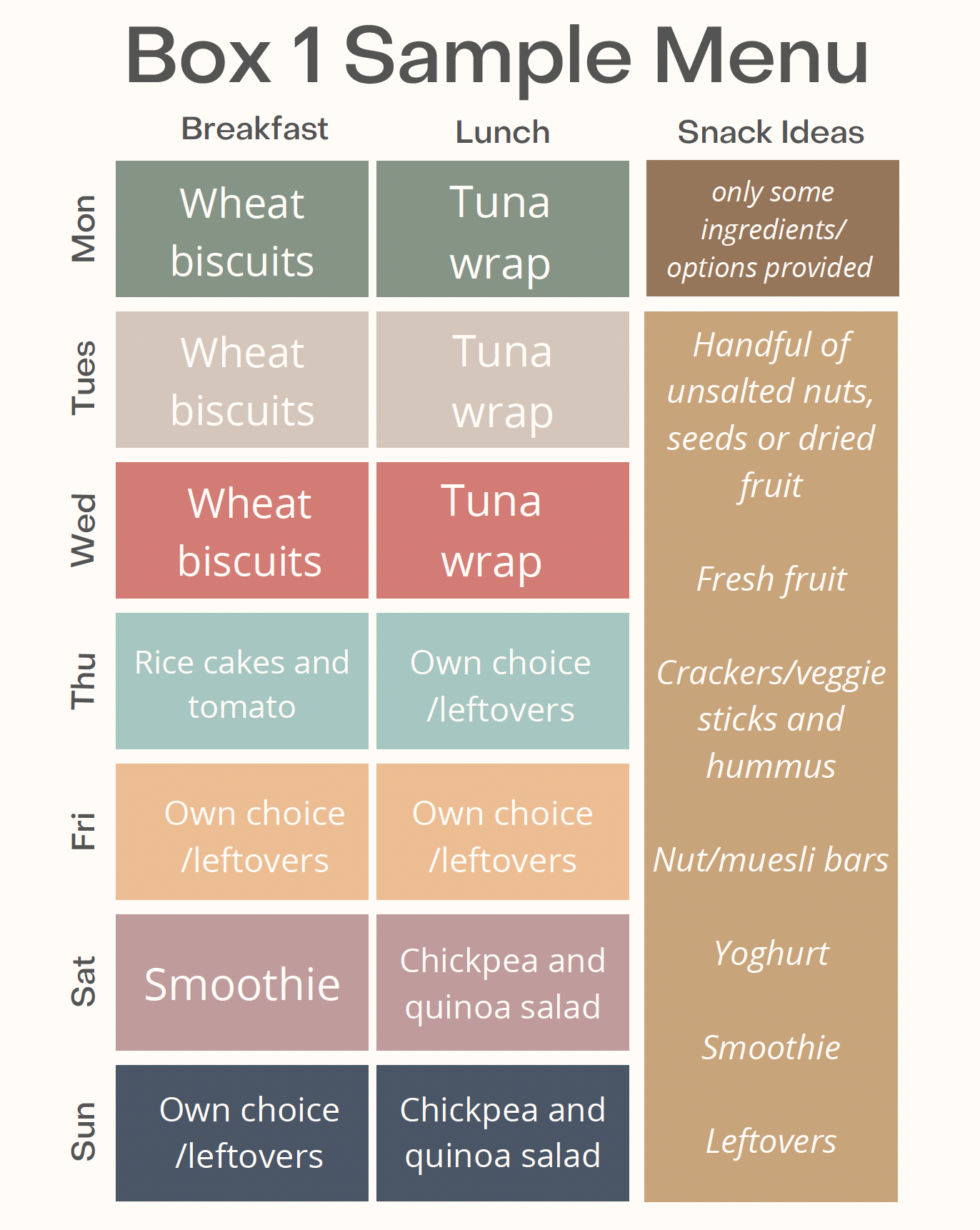


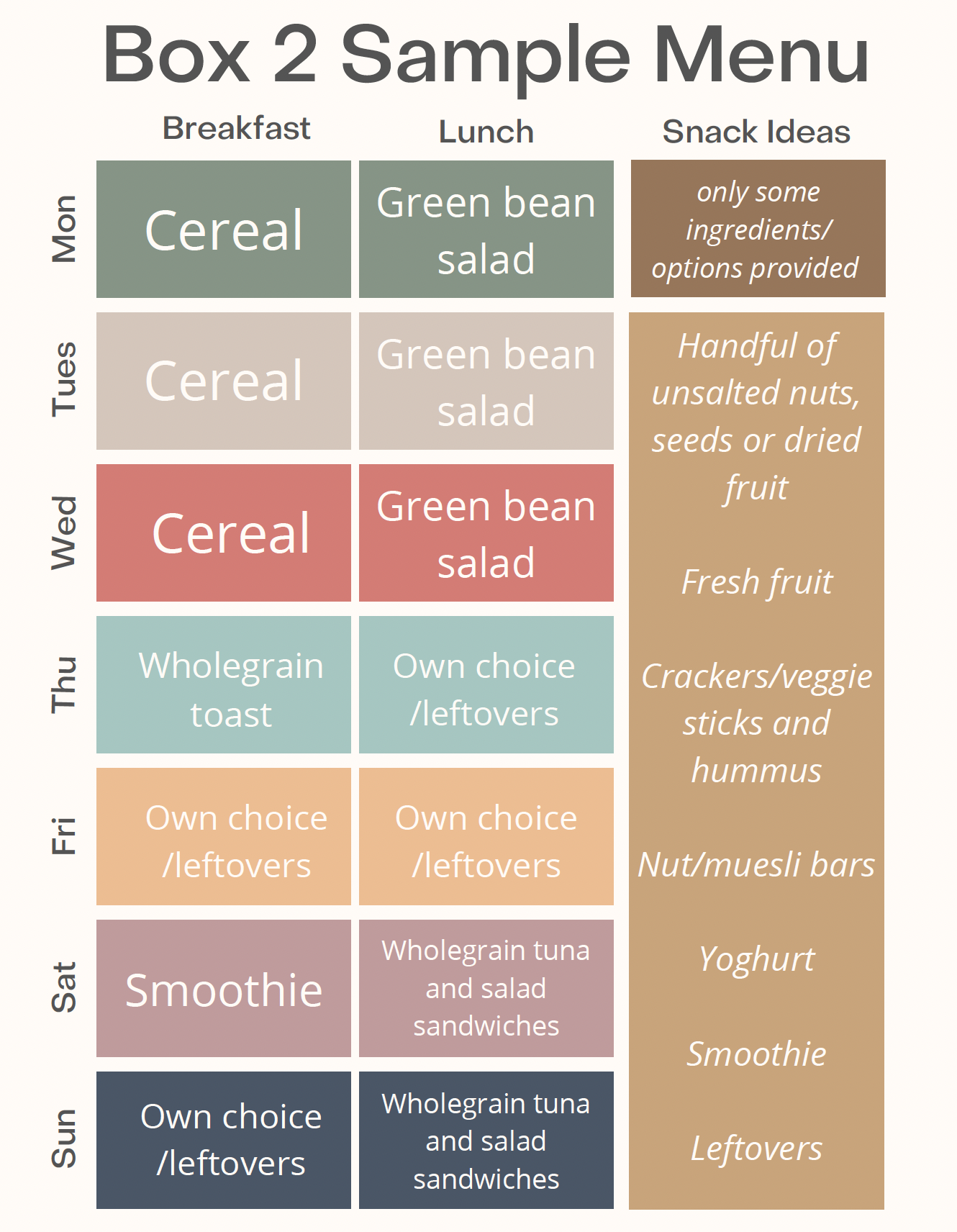


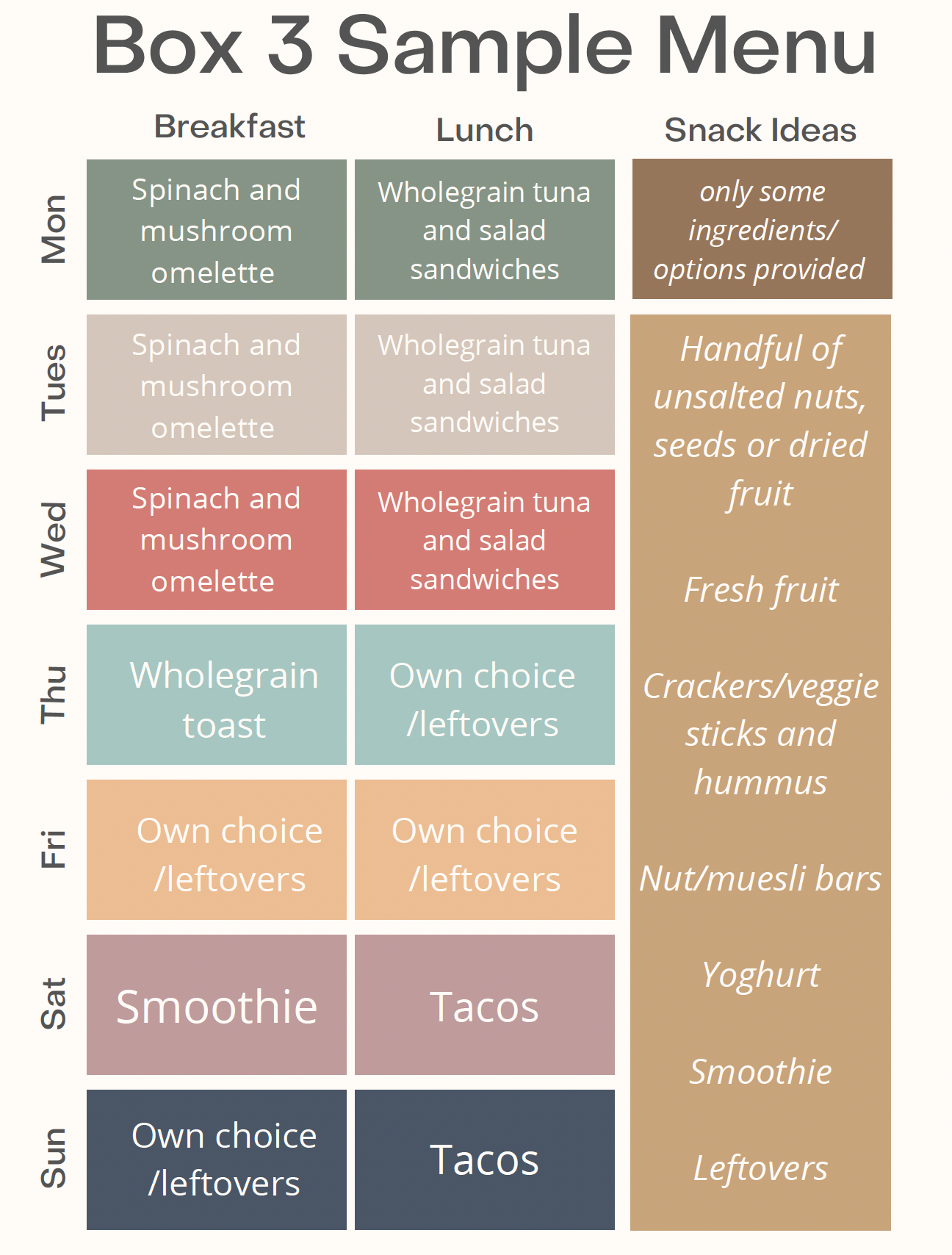


Example of dinner meals provided over one week:


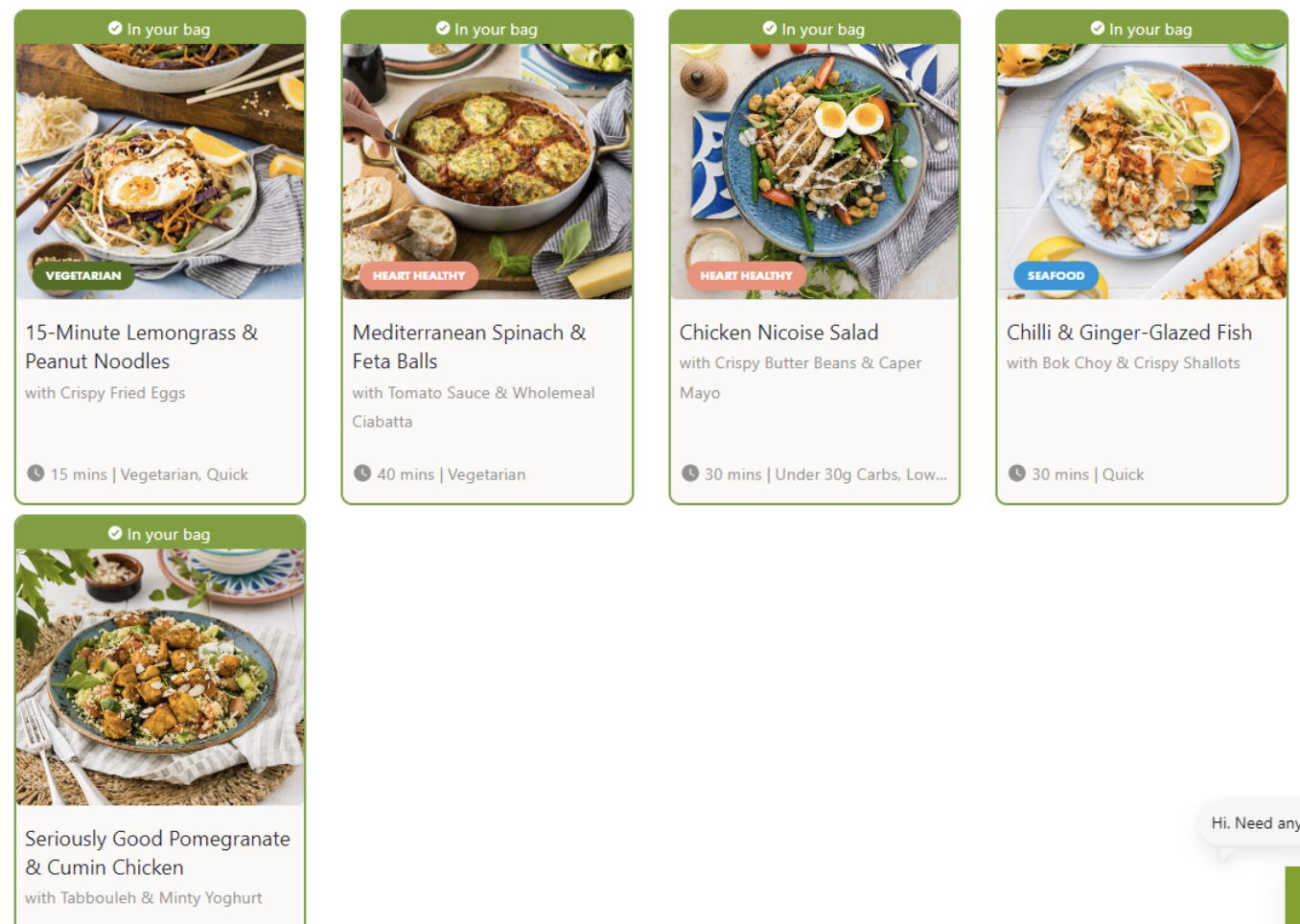

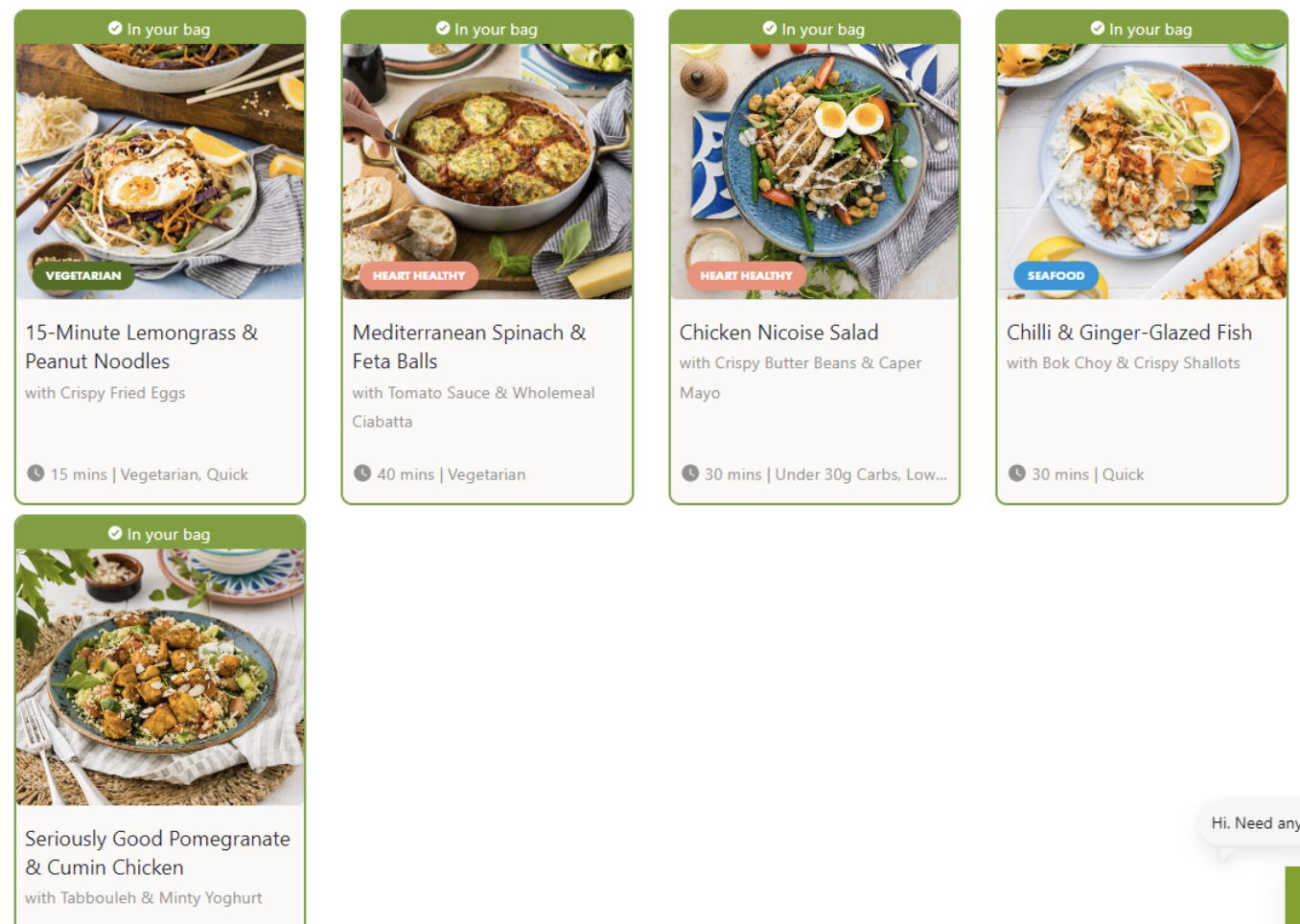

Supplement: Supplementary file 1 [file Table_1.DOCX]
