# Supplementary material for: Development of an Aotearoa New Zealand adapted Mediterranean dietary pattern and Kai/food basket for the He Rourou Whai Painga randomised controlled trial
Source: Front Nutr. 2024 Jul 26;11:1382078. doi: 10.3389/fnut.2024.1382078 (PMC11311200; doi:10.3389/fnut.2024.1382078)
Supplement: Supplementary file 2 [file Table_2.DOCX]

**Supplementary file 2.** List of industry partners and products supplied within He Rourou Whai Painga.

|  | Company | Product(s) to be provided |
| --- | --- | --- |
| 1 | Chalmers Organics Ltd | Tofu |
| 2 | Comvita New Zealand Ltd | Honey |
| 3 | Firstlight Foods Ltd | Ultimate kiwi mince |
| 4 | Goodman Fielder New Zealand Ltd | Molenburg original bread |
| 5 | Little Beauties Ltd | Air dried kiwi fruit |
| 6 | Meadow Mushrooms | Mushrooms |
| 7 | Meat Industry Association of New Zealand^1^ | Red meat (beef and lamb) |
| 8 | Moana New Zealand | Seafood |
| 9 | Olives New Zealand^2^ | Olive oil |
| 10 | Pacific Harvest | Kombu seaweed; Kelp flavoured seaweed; Kelp plain seaweed; Wakame wild harvested seaweed |
| 11 | Seadragon Marine Oils Ltd | SeaDragon SmartPac oil |
| 12 | Superb Herb | Herbs |
| 13 | Torere Macadamias | Dukkah, macadamia nuts |
| 14 | Waikōkopu Grove and Orchard | Olive oil |
| 15 | Zespri International Ltd | Kiwi fruit |
| 16 | Kristy’s Cereal Cakes | Brown rice cakes |
| 17 | Fonterra | Lite milk |
| 18 | Pics Peanut Butter | Peanut Butter |

^1^ AFFCO Limited; Alliance Group Limited; Silver Fern Farms; Greenlea Premier Meats; Auckland Meat Processors Limited; Taylor Preston Limited; Prime Range Meats Limited.

^2^ Leafyridge Olives; Dali; Noble Estate; Kapiti Olives; Loopline; Rata Olives; Blue Earth; Lot Eight; Olea Estate; The Village Press; Telegraph Hill; Kakariki Olives; Olivo; Old French Road; Mystery Valley Produce; Koru Olives; Fantail Grove
